# Development of an activity-based probe for acyl-protein thioesterases

**DOI:** 10.1371/journal.pone.0190255

**Published:** 2018-01-24

**Authors:** Megan Garland, Christopher J. Schulze, Ian T. Foe, Wouter A. van der Linden, Matthew A. Child, Matthew Bogyo

**Affiliations:** 1 Cancer Biology Program, Stanford University School of Medicine, Stanford, California, United States of America; 2 Department of Pathology, Stanford University School of Medicine, Stanford, California, United States of America; 3 Department of Chemical and Systems Biology, Stanford University School of Medicine, Stanford, California, United States of America; 4 Department of Microbiology and Immunology, Stanford University School of Medicine, Stanford, California, United States of America; Scripps Research Institute, UNITED STATES

## Abstract

Protein palmitoylation is a dynamic post-translational modification (PTM) important for cellular functions such as protein stability, trafficking, localization, and protein-protein interactions. S-palmitoylation occurs via the addition of palmitate to cysteine residues via a thioester linkage, catalyzed by palmitoyl acyl transferases (PATs), with removal of the palmitate catalyzed by acyl protein thioesterases (APTs) and palmitoyl-protein thioesterases (PPTs). Tools that target the regulators of palmitoylation–PATs, APTs and PPTs–will improve understanding of this essential PTM. Here, we describe the synthesis and application of a cell-permeable activity-based probe (ABP) that targets APTs in intact mammalian cells and the parasite *Toxoplasma gondii*. Using a focused library of substituted chloroisocoumarins, we identified a probe scaffold with nanomolar affinity for human APTs (HsAPT1 and HsAPT2) and synthesized a fluorescent ABP, JCP174-BODIPY TMR (JCP174-BT). We use JCP174-BT to profile HsAPT activity *in situ* in mammalian cells, to detect an APT in *T*. *gondii* (TgPPT1). We show discordance between HsAPT activity levels and total protein concentration in some cell lines, indicating that total protein levels may not be representative of APT activity in complex systems, highlighting the utility of this probe.

## Introduction

Post-translational modification (PTM) of proteins enables diversification of function beyond the raw coding capacity of the genome. PTMs alter the functional state of proteins, often serving as switch-like modifications that regulate transduction of intracellular signals [1]. As such, PTMs play integral roles in a range of processes including the maintenance of homeostasis [2], the cellular response to stress [3], and even host-pathogen interactions [4]. Proteins responsible for the addition and removal of PTMs, so-called writers and erasers, regulate the specificity of these modifications.

S-palmitoylation is the post-translational covalent addition of palmitic acid (a saturated 16-carbon acyl chain) onto a protein-associated cysteine residue via a thioester linkage [5,6]. One function of protein palmitoylation is to generate a lipid anchor, with the acyl chain inserting into the lipid bilayer of cell membranes to retain the palmitoylated protein close to the lipid membrane surface. In addition to its function in membrane protein organization and localization, palmitoylation affects protein stability, trafficking and protein-protein interactions [5,7]. Unlike other lipid-based PTMs such as myristoylation, palmitoylation is reversible and considered to be dynamic [8]. The addition of palmitate onto protein substrates is catalyzed by a family of enzymes called palmitoyl acyl transferases (PATs), with its removal coordinated by acyl protein thioesterases (APTs) and palmitoyl-protein thioesterases (PPTs) [5]. APTs are located in the cytosolic compartment, while PPTs localize to the lysosome and are thought to play a critical role in the turnover of palmitoylated proteins.

Modern chemical proteomic techniques and small-molecule tools have enabled functional studies of this important signaling-associated PTM. Mass-spectrometry-based global profiling of palmitoylated proteins has been achieved through metabolic incorporation of orthogonal palmitic acid analogs in live cells [8,9]. In addition to this systems-based approach, small-molecules have been used to dissect the function of the enzymes involved in the regulation of palmitoylation. For example, the small-molecule inhibitor palmostatin B (palmo B) has been used to specifically link the activity of human acyl protein thioesterase 1 (HsAPT1) with the oncogenic phenotype of H-RasG12V-transformed fibroblasts [10], and a small-molecule acyl protein thioesterase 2 (APT2) inhibitor was shown to rescue mislocalization of the scaffolding protein Scribble and increase Scribble *S*-palmitoylation upon transformation with the transcription factor Snail [11]. Using a similar small-molecule approach, we demonstrated that the *Toxoplasma gondii* orthologue of HsAPT1 (TgPPT1) is the target of a substituted chloroisocoumarin [12] and its inhibition in extracellular *T*. *gondii* tachyzoites enhanced their ability to invade host cells, highlighting a critical role for this PTM in establishing the initial point of contact between the parasite and its host cell. Additionally, a general reporter substrate for depalmitoylases was recently used to uncover signaling pathways that regulate dynamic APT activity *in situ* [13]. Although these small-molecules have proven to be valuable tools, many questions remain about the full repertoire of depalmitoylases in cells, as well as their subcellular localization and preferred substrates.

Activity-based probes (ABPs) are small-molecules functionalized with a reporter or affinity-based tag that can be used to directly monitor the enzyme activity of a given target or set of targets in complex proteomes, live cells and organisms [14]. The ABP labels target enzymes by formation of an activity-dependent covalent bond. For example, fluorophosphonate rhodamine (FP-rho) is a fluorescent ABP that exhibits broad-spectrum reactivity for serine hydrolases. This probe has been used to profile the activity of serine hydrolases in numerous experimental setups [15,16]. ABPs can be used for a wide range of chemical biology approaches, and covalent small-molecule inhibitors can often be converted into ABPs.

Previously, we found that a substituted chloroisocoumarin inhibitor covalently inhibits the *T*. *gondii* depalmitoylase TgPPT1 [12]. We therefore performed a screen of structurally related chloroisocoumarins to identify lead scaffolds for the human depalmitoylases HsAPT1 and HsAPT2. This led to the development of a new fluorescent probe for depalmitoylase activity. We demonstrate that this new tool is both selective for this enzyme class and cell-permeant, capable of labeling of both HsAPT1 and HsAPT2 *in vitro* and in live mammalian cells. It also specifically targets TgPPT1 in live *T*. *gondii* tachyzoites. In a proof-of-concept demonstration of its utility, we tested the relationship between HsAPT activity and the metastatic potential of oncogenically transformed mammalian cells. We observed an overall increase in HsAPT activity in oncogenically transformed cell lines as compared to primary cell lines. Additionally, we compared three pairs of cancer cell lines derived from the same tissue type with both high and low metastatic potential. In two of the three pairs, we observed an inverse correlation between ABP-labeled HsAPT activity and the reported metastatic potential of the cell lines. Further, total protein levels did not always correlate with APT activity measured by the ABP, indicating that tools to specifically measure enzyme activity may provide relevant information about the regulation of their activity that cannot be obtained from measures of total protein levels.

## Results

### Substituted chloroisocoumarins inhibit HsAPT1 and HsAPT2

**Fig 1:**
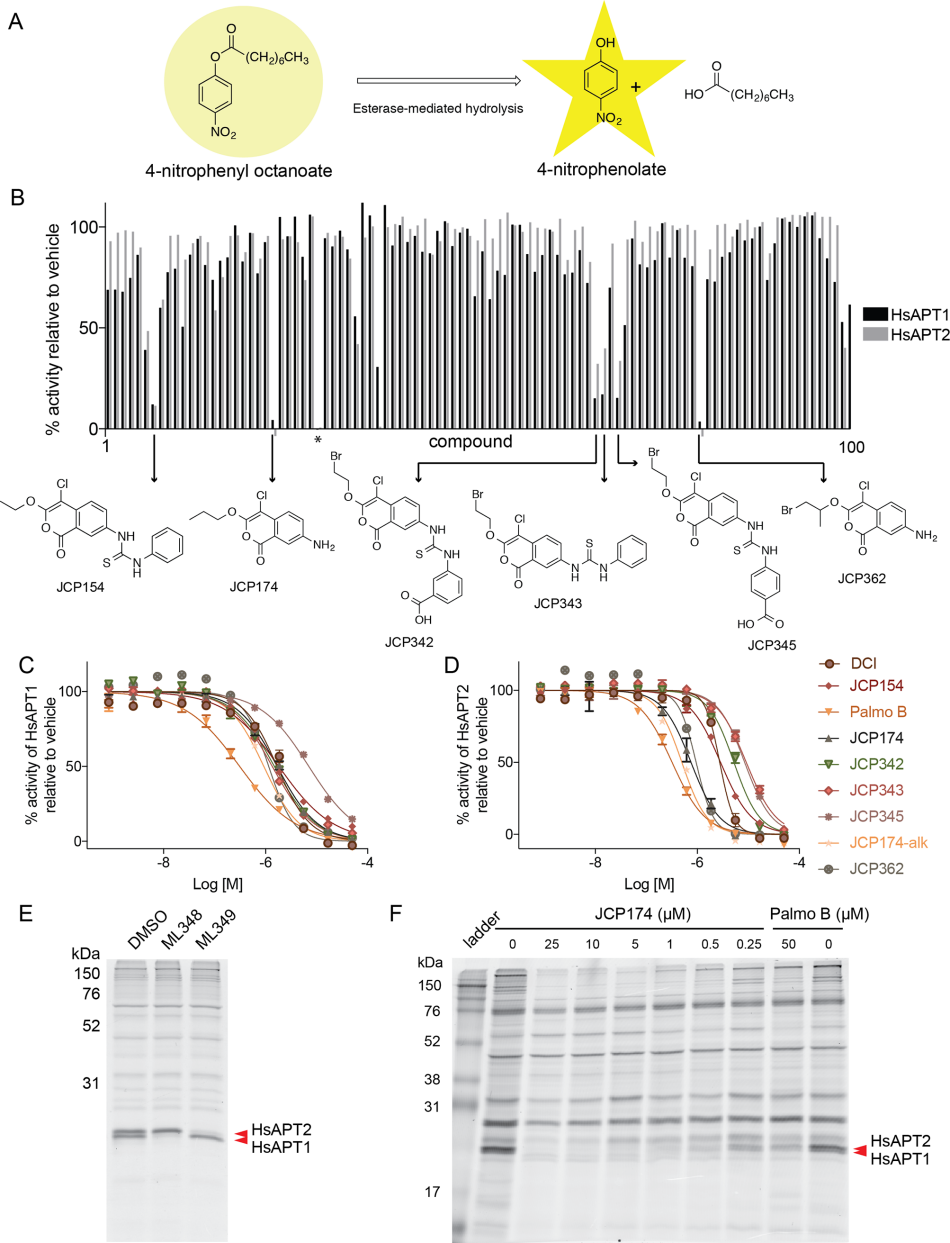
Chloroisocoumarins inhibit HsAPT1 and HsAPT2. A, 4-NPO biochemical esterase activity assay schematic. 4-nitrophenyl octanoate is converted to 4-nitrophenolate (yellow) via esterase-mediated hydrolysis and measured at 401 nm by UV-Vis spectroscopy. B, Histogram of the results of the 4-NPO assay screen with a substituted chloroisocoumarin library. Residual esterase activity of HsAPT1 and HsAPT2 is shown for each compound. Structures of potent compounds are highlighted. * indicates dichloroisocoumarin (DCI). C, D, Dose-response curves for the chloroisocoumarin hits against HsAPT1 (C) and HsAPT2 (D) plotting residual activity versus molar concantration (M). E, ABP competition labeling with the pan-serine reactive ABP FP-rho. U-2OS cells were pre-treated with DMSO control, 50 μM ML348, or 50 μM ML349 before labeling with 0.5 μM FP-rho for 5 minutes. Samples were resolved by SDS-PAGE and visualized for fluorescent probe signal with a flatbed scanner. Carets indicate HsAPT1 and HsAPT2. F, ABP competition labeling with the pan-serine reactive ABP FP-rho. Intact cells were pre-treated with different concentrations of JCP174 or palmostatin B (Palmo B) before labeling with FP-rho. Samples were prepared as in (E). Carets indicate doublet of interest at ~25 kDa identified as HsAPT1 and HsAPT2.

We recently performed a phenotypic screen to identify small-molecule modulators of host cell invasion by the apicomplexan parasite *T*. *gondii* [12,17]. The small-molecules in this directed screen were expected to covalently modify protein targets in an activity-dependent manner [18]. Unexpectedly, this screen not only identified inhibitors of parasite invasion, but also compounds that enhanced host cell invasion. Enhancer compounds were all structurally similar substituted chloroisocoumarins [12]. Using an alkynylated version of the lead enhancer compound (JCP174), we identified the phenotypically relevant target of the enhancer phenotype as the previously uncharacterized thioesterase TgPPT1 [12]. TgPPT1 is the orthologue of the human depalmitoylase HsAPT1, sharing 33% identity by BLAST analysis. On the basis of this similarity, we reasoned that substituted chloroisocoumarins would likely inhibit the activity of the human depalmitoylases HsAPT1 and HsAPT2 and potentially serve as novel scaffolds to generate selective covalent probes. Therefore, we screened a focused library of substituted chloroisocoumarins to identify compounds that inhibited HsAPT1 and HsAPT2 activity. We expressed recombinant HsAPT1 and HsAPT2 with an N-terminal His_6_ tag in *E*. *coli* and purified the proteins using nickel affinity chromatography (to a final purity of >95%, as shown in S1 Fig). To measure the enzymatic activity of recombinant HsAPT1 and HsAPT2, we used the previously reported substrate, 4-nitrophenyl octanoate (4-NPO), in which enzymatic cleavage of the ester linkage can be measured by UV/Vis spectroscopy upon release of 4-nitrophenolate [10] (Fig 1A). Using this activity readout, we performed our screen with recombinant HsAPT1 and HsAPT2 using a focused library of 100 substituted chloroisocoumarins (Fig 1B). Compounds that produced >50% inhibition of either HsAPT1 or HsAPT2 activity relative to the DMSO control at 10 μM were then re-screened across a range of concentrations to determine *in vitro* IC_50_ values (Fig 1C and 1D). Compounds with IC_50_ values <10 μM for both HsAPT1 and HsAPT2 were scored as hits. According to these criteria, our screen achieved a hit rate of 7% (Table 1). The chloroisocoumarin JCP174 exhibited the greatest potency towards both HsAPTs, with IC_50_ values of 1.7 μM and 0.75 μM against HsAPT1 and HsAPT2, respectively (Table 1). We next tested the potency of JCP174 for both enzymes with increasing preincubation times from 30 minutes to 6 hours. The potency of JCP174 increased with longer pre-incubation times for both targets, indicating an irreversible covalent mechanism (S1 Table). In contrast, the published inhibitor of HsAPTs, palmo B, exhibited decreased potency with longer preincubation times, as expected given its reversible mechanism of inhibition and stability in aqueous solutions [10] (S1 Table).

**Table 1:** IC_50_ values of substituted chloroisocoumarins for rHsAPT1 and rHsAPT2 in the 4-NPO esterase assay.

Compound	HsAPT1 IC_50_ (μM)	HsAPT2 IC_50_ (μM)
**DCI**	1.7	2.7
**Palmostatin B**	0.31	0.32
**JCP154**	1.8	2.9
**JCP174**	1.7	0.75
**JCP342**	1.5	5.2
**JCP343**	1.4	9.1
**JCP345**	7.1	8.5
**JCP362**	1.2	0.95
**JCP174-alk**	0.93	0.50
**JCP174-BODIPY TMR**	3.0	1.8

To determine the selectivity of JCP174 and assess engagement with HsAPT1 and HsAPT2 in live mammalian cells, we performed ABP competition labeling experiments. As chloroisocoumarins are reported to be serine hydrolase inhibitors [12,19–21], with dichloroisocoumarin (DCI) being the prototypical member of this inhibitor class, we selected the broad-spectrum serine hydrolase ABP, FP-rho [16] for these experiments. We first determined whether this broad-spectrum ABP, FP-rho, labeled HsAPT1 and HsAPT2. To identify ABP-labeled protein species representing HsAPT1 and HsAPT2 in gel-based assays, we used previously published selective and reversible inhibitors of HsAPT1 and HsAPT2, ML348 and ML349 [22]. As these piperazine amides are highly selective for binding to either HsAPT1 (ML348) or HsAPT2 (ML349), competition for target engagement between FP-rho and these compounds allowed us to identify each enzyme in the labeling pattern of this broad-spectrum probe. We pretreated mammalian U-2 OS cell lysates with 50 μM each of ML348 and ML349, or DMSO control for 1 hour, then labeled with FP-rho for 5 minutes. This short probe labeling time was chosen due to the reversible mechanism of these selective inhibitors, which also precludes their use as ABPs. Labeling was assessed by measuring fluorescent signal associated with the resolved proteins species in-gel using a flatbed scanner. Compared to the DMSO control, in which FP-rho strongly labels two closely migrating species of approximate 25 kDa molecular weight, ML348 competed for labeling of the lower protein of the doublet, while ML349 competed for the higher protein in this doublet (Fig 1E). These data identify HsAPT1 as the lower band of the doublet and HsAPT2 as the upper band in the doublet at 25 kDa (Fig 1E), in accordance with previous reports [22]. To determine whether JCP174 could also compete for labeling of HsAPT1 and HsAPT2, we pretreated U-2 OS cells with JCP174 for 1 hour, washed the cells and prepared cell lysates that were then labeled with FP-rho to assess residual activity of the hydrolase targets. JCP174 also competed with FP-rho for the labeling of both HsAPT1 and HsAPT2 (Fig 1F). Further, palmo B [10] competed for the same two species (Fig 1F). These data demonstrate that JCP174, the most potent hit from our screen, acts via an irreversible covalent mechanism and effectively inhibits HsAPT1 and HsAPT2.

### The ABP JCP174-BODIPY TMR labels HsAPT1 and HsAPT2 *in vitro*

**Fig 2:**
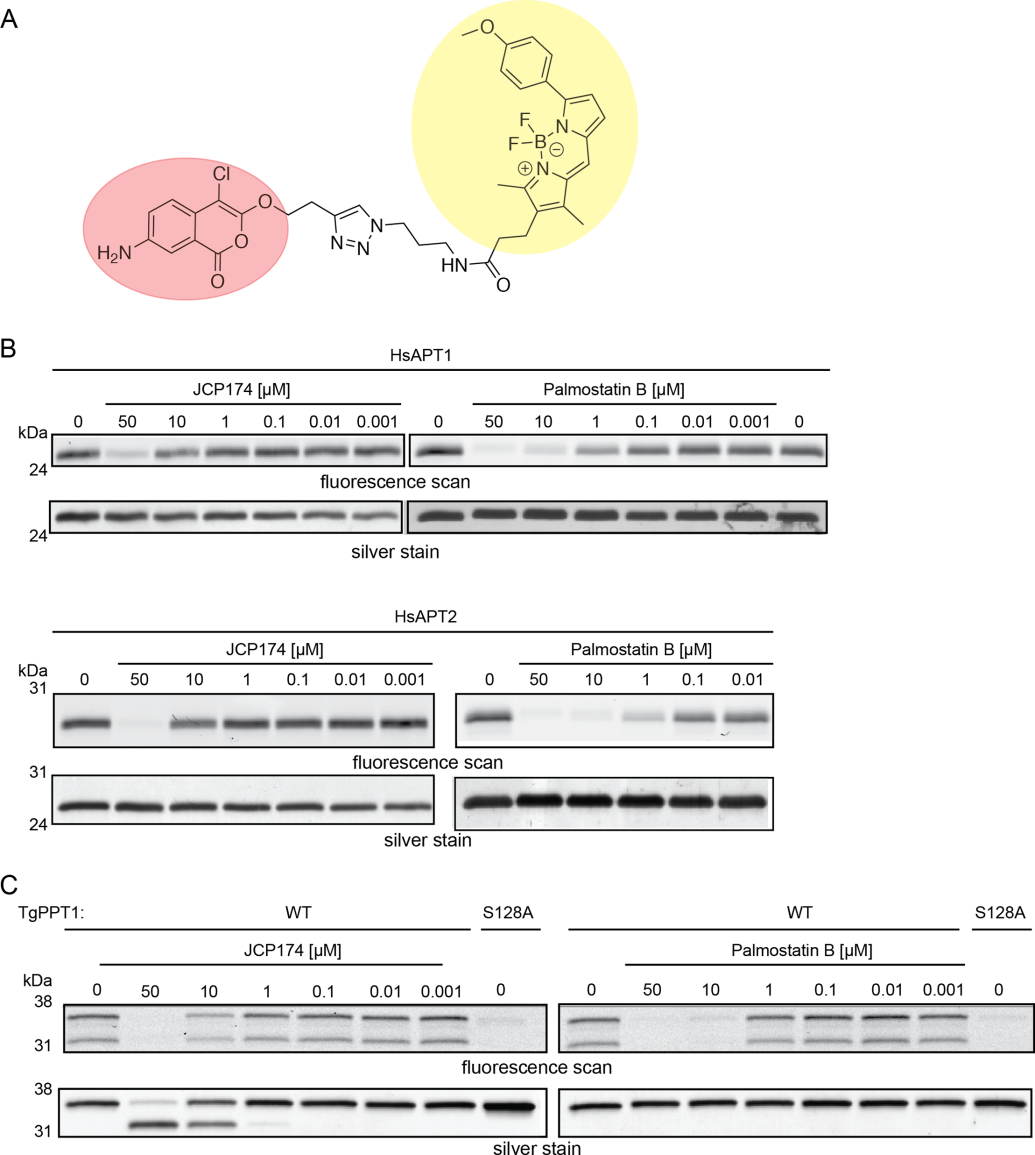
JCP174-BT labels HsAPT1, HsAPT2 and TgPPT1 *in vitro*. A, Structure of JCP174-BODIPY TMR (JCP174-BT) composed of chloroisocoumarin small-molecule JCP174 (red) and BODIPY TMR fluorophore (yellow). B, C, In-gel ABP competition labeling with JCP174-BT. (B) Purified recombinant HsAPT1 or HsAPT2 was pre-treated with different concentrations of JCP174, palmostatin B, or DMSO (0) before labeling with JCP174-BT. Samples were resolved by SDS-PAGE and fluorescent probe signal visualized using a flatbed scanner (upper panel), with loading assessed via silver stain (lower panel). (C) Purified recombinant TgPPT1 WT and active site mutant (S128A) were treated with JCP174, palmostatin B or DMSO (0) and labeled with JCP174-BT. Samples were analyzed as in (B).

Following the identification of JCP174 as the lead inhibitor for HsAPT1 and HsAPT2, we sought to convert this compound into an ABP. We previously synthesized an alkynylated version of JCP174 (JCP174-alk) [12]. Functionalization of JCP174 with an alkyne tag did not reduce its potency for either HsAPT1 or HsAPT2, with IC_50_ values of 0.925 μM and 0.498 μM respectively (Fig 1C and 1D, Table 1). We then used JCP174-alk to synthesize a fluorescent ABP in a one-step click reaction with an azide-BODIPY TMR fluorophore (543 nm excitation, 569 nm emission; Fig 2A, S2 Fig). Modification of JCP174-alk with the BODIPY TMR tag only moderately reduced its potency, with IC_50_ values for HsAPT1 and HsAPT2 of 3.0 μM and 1.8 μM, respectively (Table 1). This confirmed that functionalization with the fluorophore did not abolish affinity towards the target enzymes (S3 Fig).

We first determined if JCP174-BODIPY TMR (JCP174-BT), was able to covalently label recombinant HsAPT1, HsAPT2, and TgPPT1. We observed dose-dependent competition with the parent compound, JCP174, and palmo B in an ABP competition assay with JCP174-BT (Fig 2B and 2C). We also observed that covalent modification of TgPPT1 by the parent compound JCP174 resulted in a downward shift in the migration of the species when resolved by SDS-PAGE (Fig 2C). Even though the protein is a single band by silver stain, we observed two fluorescent bands upon probe labeling. This may be due to the fact that chloroisocoumarins have two sites of attack by serine and histidine residues, rendering two different migratory species. At higher concentrations of JCP174 treatment, only the lower migration species is observed, indicating that this species may be the final version after cyclization and loss of chlorine (Fig 2C). A change in the migration of TgPPT1 following covalent modification by small-molecules has been previously reported [23]. To confirm the specificity of JCP174-BT for the active site of TgPPT1 and activity-dependence of probe labeling, we compared in-gel labeling of the wild-type TgPPT1 (TgPPT1^WT^) versus a catalytically dead TgPPT1 mutant where the active site serine was mutated to an alanine (TgPPT1^S128A^) [12]. JCP174-BT covalently modified TgPPT1^WT^, but did not label the active site mutant, TgPPT1^S128A^ (Fig 2C). Together, these data show that the JCP174-BT probe retains potency for HsAPT1 and HsAPT2, and covalently modifies the active site serine.

### JCP174-BT labels depalmitoylases in intact parasites and mammalian cells

**Fig 3:**
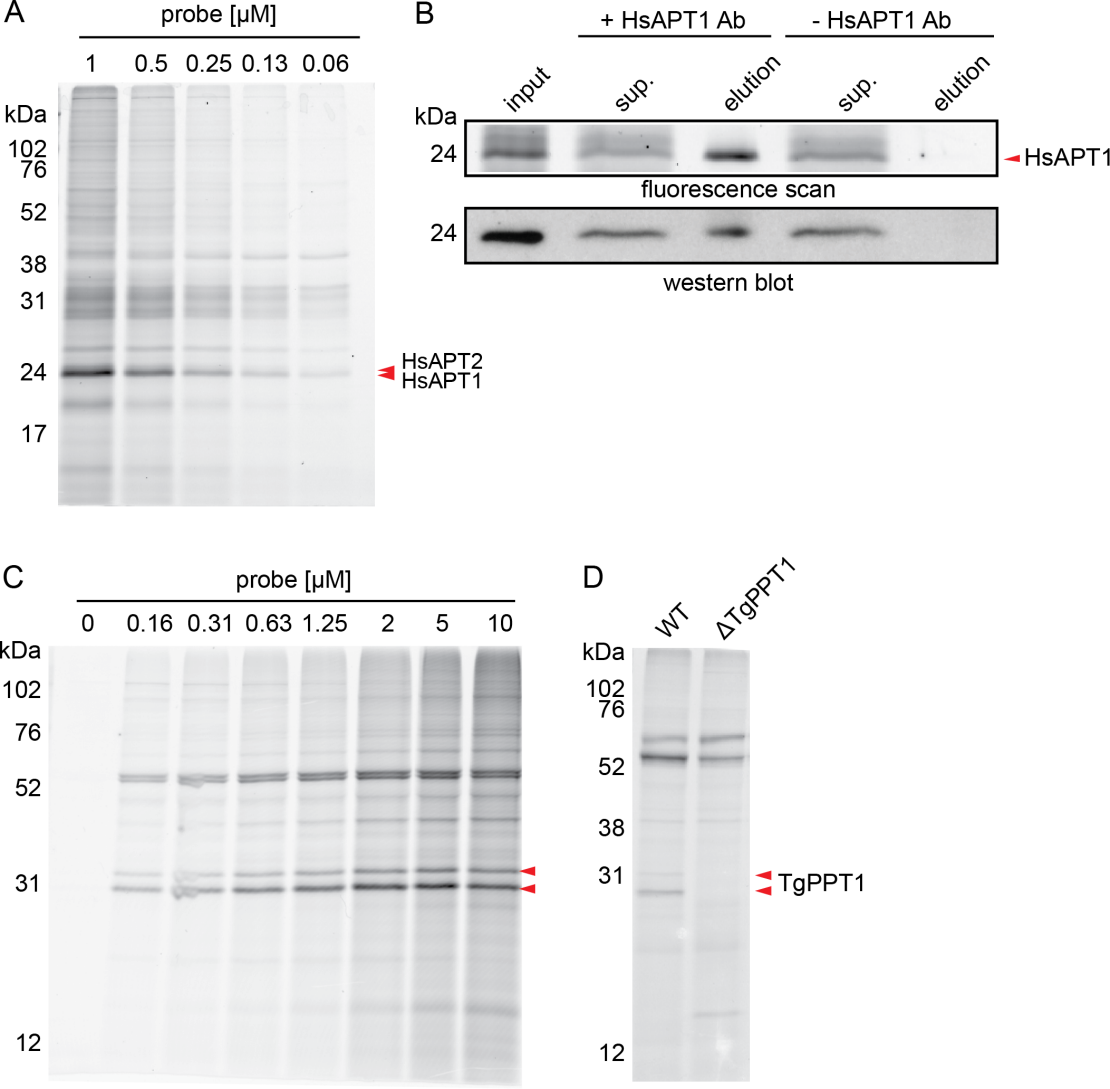
JCP174-BT labels HsAPT1, HsAPT2 and TgPPT1 in intact cells. A, Intact U-2 OS mammalian cells were labeled with different concentrations of JCP174-BT, washed to remove unbound probe, and lysed. Lysates were resolved by SDS-PAGE with fluorescent probe labeling visualized using a flatbed scanner. Carets indicate doublet of interest ~25 kDa previously identified as HsAPT1 and HsAPT2. B, Intact U-2 OS mammalian cells were labeled with JCP174-BT, washed to remove unbound probe, and lysed. HsAPT1 was immunoprecipitated from the lysate, with input, supernatant, and elution samples resolved by SDS-PAGE. Fluorescent probe signal was visualized via flatbed scanner (upper panel). HsAPT1 identity was confirmed by western blot (lower panel). The red caret indicates HsAPT1 species on the fluorescent scan. C, Intact *T*. *gondii* tachyzoites were labeled with different concentrations of JCP174-BT, washed to remove unbound probe and lysed. Lysates were resolved by SDS-PAGE with fluorescent probe labeling visualized using a flatbed scanner. Carets indicate bands of interest ~31 kDa. D, Intact wild-type *T*. *gondii* or ΔTgPPT1 tachyzoites were labeled with JCP174-BT as in (C). Carets indicate species corresponding to TgPPT1 in labeling of wild-type tachyzoites.

After verifying the ability of the probe to label recombinant enzymes *in vitro*, we tested its ability to label native depalmitoylases in intact cells. We observed strong labeling of the two species that resolved as a closely migrating doublet around 25 kDa (Fig 3A) that we previously identified as HsAPT1 and HsAPT2 (see Fig 1E). To further confirm that the lower migrating species of the probe-labeled doublet was HsATP1, we performed an immunoprecipitation (IP) using an anti-HsAPT1 antibody (ProteinTech). We observed a strongly labeled doublet around 25 kDa in the input, with the lower species depleted in the unbound IP supernatant, and this same lower molecular weight species was present in the IP elution (Fig 3B). We confirmed HsAPT1 to be the fluorescently labeled species by western blot (Fig 3B). These data confirmed that JCP174-BT covalently modified native HsAPT1 in live mammalian cells. While it is highly likely that the labeled upper band is APT2, we were unable to find an antibody that would allow direct immunoprecipitation to confirm this. Furthermore, both the APT1 and APT2 inhibitors failed to compete for any of the species labeled by the JCP174-BT probe likely because of the reversible nature of the molecules and the slower rate of covalent modification by JCP174-BT compared to FP-rho.

In addition to labeling HsAPT1 and HsAPT2 in intact cells, we tested whether JCP174BT could label other depalmitoylases *in situ*. We labeled live extracellular *T*. *gondii* tachyzoites with increasing concentrations of JCP174-BT, washed the parasites to remove excess probe, lysed them, and resolved soluble lysates by SDS-PAGE. We observed two strongly labeled species around 31 kDa in size (Fig 3C). To determine which of the labeled species visualized in the lysate is TgPPT1, we used a previously generated *T*. *gondii* TgPPT1 knockout strain (ΔTgPPT1) [12]. Comparison of the probe labeling of wild-type or ΔTgPPT1 parasite strains revealed that the doublet at ~31 kDa in the wild-type strain was absent in the ΔTgPPT1 knockout strain, confirming the identity of these two species as TgPPT1 (Fig 3D). This was also consistent with the doublet species observed for recombinant enzyme preparations of TgPPT1 labeled with JCP174-BT (Fig 2C). Together, these data show that JCP174-BT is cell-permeant and covalently modifies TgPPT1 and HsAPT1 in intact cells.

### JCP174-BT profiles APT activity in intact mammalian cells and *T*. *gondii* parasites

**Fig 4:**
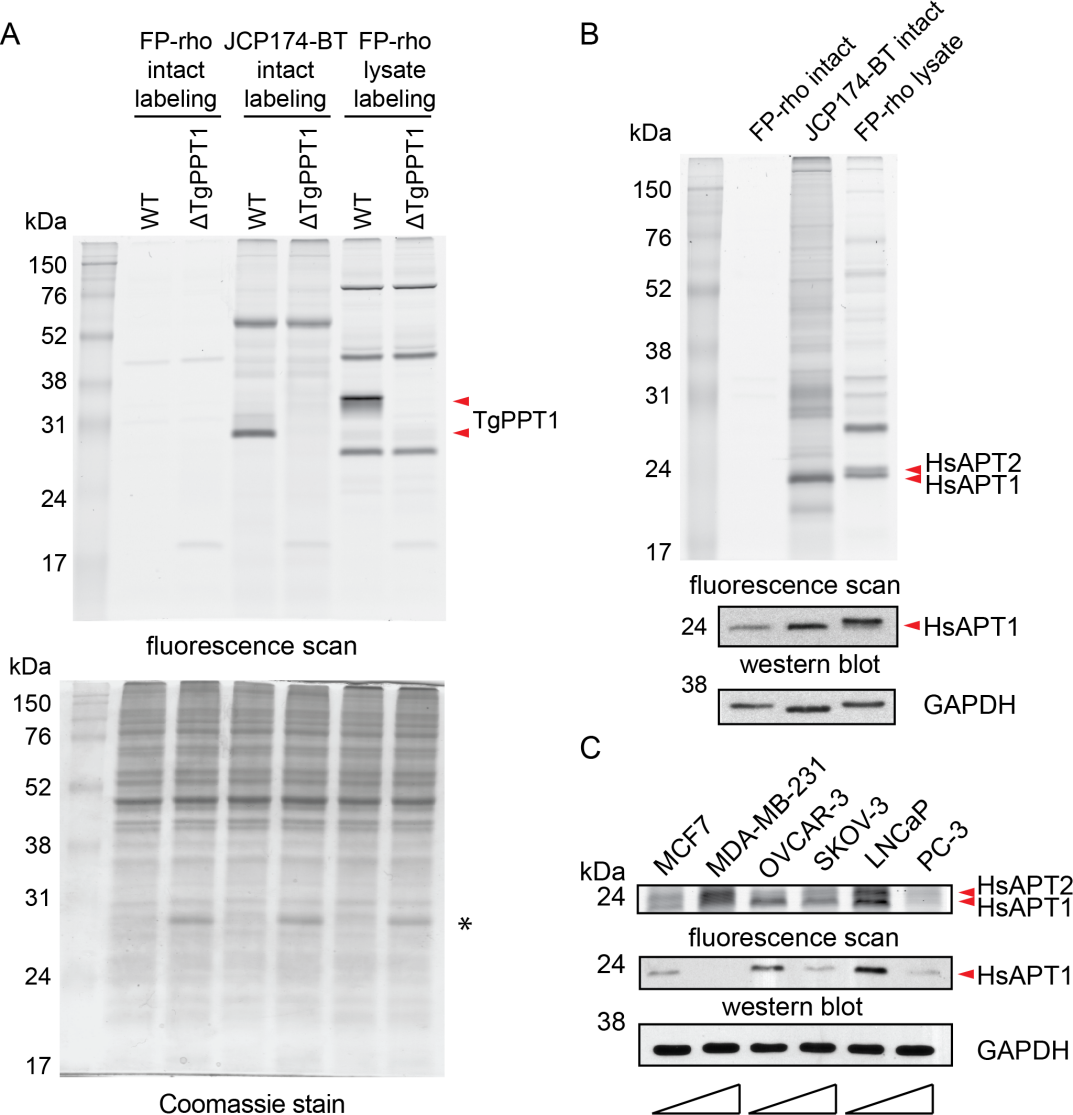
JCP174-BT profiles APT activity in intact mammalian cells and *T*. *gondii* parasites. A, Intact wild-type *T*. *gondii* or ΔTgPPT1 tachyzoites were labeled with JCP174-BT or FP-rho, washed to remove unbound probe and lysed. *T*. *gondii* tachyzoite lysate was labeled with FP-rho. Lysates were resolved by SDS-PAGE with fluorescent probe labeling visualized using a flatbed scanner. Carets indicate species corresponding to TgPPT1 in labeling of wild-type tachyzoites. Asterisk (*) indicates m-Cherry expressed in ΔTgPPT1 parasites. B, Intact U-2 OS cells were labeled with JCP174-BT or FP-rho, washed to remove unbound probe, and lysed. U-2 OS lysate was labeled with FP-rho. Lysates were resolved via SDS-PAGE. Fluorescent signal was visualized with a flatbed scanner (fluorescence scan), with the coomassie stain of the same gel shown to indicate loading. Carets indicate the species corresponding to HsAPT1 and HsAPT2. C, Intact mammalian cells were labeled with JCP174-BT and processed as in (A). Pairs of oncogenic cell lines from derived from three tissue types (carcinomas of the breast, ovary and prostate) were chosen to contrast low metastatic/aggressive potential (MCF7, OVCAR-3, and LNCaP) versus high metastatic potential (MDA-MB-231, SKOV-3, and PC-3) [25]. For each oncogenic pair, wedges indicate low to high metastatic potential. Fluorescent signal was visualized with a flatbed scanner (top panel). Total HsAPT1 protein level was visualized by western blot (middle panel). GAPDH was used as a loading control (lower panel).

To demonstrate the utility of JCP174-BT to profile APT activity, we compared its labeling to that of the broad spectrum ABP FP-rho in intact *T*. *gondii* tachyzoites and *T*. *gondii* lysates. We treated intact wild-type or ΔTgPPT1 parasites with the same concentration of FP-rho or JCP174-BT. We also labeled *T*. *gondii* lysates with the same concentration of FP-rho. When visualized by in-gel fluorescence, JCP174-BT showed strong labeling of TgPPT1 in intact cells, and this labeling was lost in the ΔTgPPT1 parasites (Fig 4A). However, FP-rho was unable to label TgPPT1 in intact parasites suggesting that it likely is not cell-permeant (Fig 4A). JCP174-BT predominantly labeled TgPPT1 and one other protein of approximately 55 kDa, while FP-rho predominantly labeled four species in cellular lysates. These data demonstrate the advantage of JCP174-BT to label intracellular depalmitoylases in intact tachyzoites.

To further demonstrate the utility of JCP174-BT, we performed the same comparison with human U-2 OS cells. Comparison of intact cell labeling with JCP174-BT, FP-rho, or lysate labeling with FP-rho revealed different fluorescent banding patterns (Fig 4B). Similar to the studies in *T*. *gondii* cells, FP-rho did not significantly label any species when used on intact cells but did show robust labeling of serine hydrolases, including HsAPT1 and HsAPT2 when added to cellular lysates (Fig 4B). In contrast, JCP174-BT labeled APT1 and APT2 in intact cells, highlighting its utility over a more broad-spectrum ABP (Fig 4B).

Palmitoylation and APTs have been linked to cancer phenotypes, with reports of small-molecule inhibition of APTs able to partially revert the H-RasG12V-dependent oncogenic phenotype [10]. Additionally, increased metastatic potential of cancers correlates with increased incorporation of exogenous palmitate onto oncogenic signaling molecules [24]. Therefore, after confirming that JCP174-BT was cell-permeant and capable of labeling HsAPT1 and HsAPT2 in intact mammalian cells, we sought to profile HsAPT activity in cancer cell lines. As palmitoylation is a dynamic PTM important in oncogenic signaling through proteins such as Ras, we hypothesized that HsAPT activity would decrease in cancer cell lines of higher metastatic potential, with the decreased activity of depalmitoylases leading to a greater number of palmitoylated oncogenic signaling molecules. To test this, we profiled HsAPT activity in three matched cancer cell line pairs representing cell lines derived from carcinoma of the breast, ovaries, and prostate. These matched pairs each contained one cancer cell line with high metastatic or invasive potential and one with low metastatic potential [24,25]. We labeled intact cells with JCP174-BT, and imaged labeling profiles in-gel as before. While there was no uniform trend among all three matched cell lines, in-gel fluorescence showed decreased HsAPT activity in cell lines of higher metastatic potential for two of the three matched pairs (Fig 4C and S4 Fig). We next wanted to determine whether HsAPT activity observed via JCP174-BT labeling correlated with total HsAPT protein expression in each of the cell lines. Using the Cancer Cell Line Encyclopedia (CCLE) RMA-normalized mRNA expression data for HsAPT1 and HsAPT2 in the matched cancer cell lines (S2 Table) [26], we confirmed the presence of HsAPT1 and HsAPT2 in each cell line tested. Via western blot, we observed higher total protein expression of HsAPT1 in each of the three cell lines with lower metastatic potential (Fig 4C and S4 Fig). Further analysis of HsAPT1 in the matched cancer cell lines indicated discordance between total protein expression of HsAPT1, as measured by western blot, and activity of HsAPT1, measured via JCP174-BT labeling (Fig 4C). The lack of correlation between protein- and activity-levels was most apparent for the MCF7/MDA-MB-231 breast cancer-derived matched oncogenic pair. Here, we observed greater HsAPT activity in the cell line with higher metastatic potential (MDA-MB-231), which was confirmed by FP-rho labeling of lysates (S4 Fig). We observed a stronger labeling of HsAPT1 in the MCF7 cell line, while HsAPT1 and HsAPT2 displayed similar activity in the MDA-MB-231 cell line. Slight discrepancies in the labeling patterns of JCP174-BT and FP-rho may be due to the fact that FP-rho labeling was performed in cell lysates, allowing this probe to access all serine hydrolases, while some serine hydrolases may be retained in inaccessible compartments in intact cell labeling. Together, these data suggest that protein expression of APTs may not be representative of APT activity within the cell and highlights the utility of JCP174-BT to profile activity of these enzymes *in situ*.

## Discussion

Acyl-protein thioesterases are key regulatory enzymes for dynamic palmitoylation, but tools to study them remain limited. We have successfully expanded the toolset available with the development of an ABP that exhibits selectivity for acyl protein thioesterase activity in two species and present proof-of-concept studies that demonstrate its ability to probe the activity of these enzymes *in vitro* and in a diverse range of cell types.

Using an alkynylated covalent inhibitor scaffold previously identified as an inhibitor of the *T*. *gondii* APT orthologue TgPPT1, we synthesized a probe via a single-step copper-catalyzed cycloaddition reaction with an azido-fluorophore. The probe labels recombinant protein *in vitro* and can access and selectively label APTs in live, intact cells. With a selective ABP tool now available for the study of depalmitoylases, it is worthwhile noting the wider potential future applications, such as for fluorescent polarization target-based small-molecule screens [27] and screening in complex lysates [28]. In particular, further functionalization of the probe with a fluorescence quencher group (such as QSY 21) would facilitate selective imaging and subcellular localization of the active pool of these enzymes in cells and potentially for *in vivo* models of health and disease [14].

APTs have been implicated in cancer, with the broad-spectrum APT inhibitor palmo B able to partially reverse the oncogenic phenotype of H-RasG12V-transformed fibroblasts [10]. A simple interpretation of these data is that APT activity contributes to the maintenance of an oncogenic cell state, and therefore might correspondingly be higher in cancer cells. We used our probe to more broadly test this, and found that APT activity in cancer cell lines was indeed higher. However, for matched pairs of cancer cell lines, overall metastatic potential inversely correlated with APT activity, indicating that although inhibition of this enzyme class may reduce oncogenic phenotypes, it may also promote overall increased metastatic propensity due to higher global palmitoylation of key signaling molecules. Further studies will be required to test this hypothesis, but our study serves to highlight the power of this probe for tracking depalmitoylase activity in intact cell systems.

## Methods

### Protein expression and purification

HsAPT1 (26.8 kDa) and HsAPT2 (26.9 kDa) were cloned into pET28a expression vectors bearing an N-terminal His_6_ tag using 5' Nde1 and 3' BamHI (HsAPT1) or HindIII (HsAPT2) restriction sites. Constructs were transformed into chemically competent *E*. *coli* BL21(DE3)pLysS cells (Lucigen, Middleton, WI) for expression. Expression was induced with 1 mM isopropyl β-d-1-thiogalactopyranoside (IPTG) for 4 hours. Cell pellets were resuspended in lysis buffer (50 mM TRIS, 500 mM NaCl, 10% glycerol, 15 mM imidazole, pH 8.8) and membranes were disrupted via sonication on ice. Lysates were clarified by centrifugation at 16,500 rpm for 30 minutes. N-terminally His_6_-tagged HsAPT constructs were purified with Ni^2+^ metal affinity chromatography. Purified protein was eluted with high imidazole elution buffer (50 mM TRIS, 150 mM NaCl, 300 mM imidazole, pH 8.8), aliquoted, rapidly frozen in liquid nitrogen, and stored at −80°C. TgPPT1^WT^ and TgPPT1^S128A^ were expressed and purified as in Child *et al*. [12].

### 4-NPO esterase activity assay

The 4-nitropenhyl octanoate (4-NPO) activity assay was modified from Dekker *et al*. [10]. Briefly, 50 μL 20 mM HEPES, 150 mM NaCl, 0.01% v/v Triton-X 100, pH 7.4 buffer with the indicated concentrations of inhibitor or buffer control was added to 96-well plates. To each well, 30 μL of recombinantly expressed enzyme diluted to 1 μM in 20 mM HEPES, 150 mM NaCl, 0.01% v/v Triton-X 100, pH 7.4 buffer was added. The inhibitor and enzyme reaction was incubated at 37°C for 30 minutes before 20 μL of 4-NPO (3.0 mM) was added to the reaction in 20 mM HEPES, 150 mM NaCl, 0.25% v/v Triton-X 100, pH 7.4 buffer. The reaction progress was measured using a Cytation 3 plate reader at 401 nm at 1-minute intervals for 40 minutes.

### Cell culture

Cell lines were a generous gift from the laboratory of S. Contag. SKOV3 (ATCC HTB-77) and U-2 OS (ATCC HTB-96) cells were cultured in McCoy’s media supplemented with 10% (v/v) fetal calf serum, 10 U/mL penicillin G, and 100 μg/mL streptomycin. PC-3 (ATCC CRL-1435) and LnCaP (ATCC CRL-1740) cells were cultured in RPMI supplemented with 10% (v/v) fetal calf serum, 10 U/mL penicillin G, and 100 μg/mL streptomycin. OVCAR-3 (ATCC HTB-161) cells were cultured in RPMI supplemented with 20% (v/v) fetal calf serum, 10 U/mL penicillin G, and 100 μg/mL streptomycin. MDA-MB-231 (ATCC HTB-26) and MCF7 (ATCC HTB-22) cells were cultured in Dulbecco's modified medium (DMEM) supplemented with 10% (v/v) fetal calf serum, 10 U/mL penicillin G, and 100 μg/mL streptomycin, and 2 mM L-glutamine. All cells were incubated at 37°C and 5% CO_2_.

### FP-rhodamine competition labeling assay

U-2 OS cells were seeded at a density of 300,000 cells per well into a 6-well plate and allowed to adhere overnight. Cells were pre-treated with different concentrations of JCP174 or palmostatin B for 1 hour at 37°C. Cells were washed with PBS and lysates were prepared by incubating pellets in TRIS-buffered saline (TBS, 50 mM TRIS, 150 mM NaCl, pH 7.4) with 0.5% NP40 for 30 minutes on ice. Lysates were clarified via centrifugation at 13,000 rpm for 30 minutes, and total protein concentration was measured via bicinchoninic acid (BCA) assay. For labeling with FP-rhodamine, 20 μg total protein was incubated with 0.5–1 μM FP-rhodamine (30× stock in DMSO) in a total volume of 15 μL for 5–30 minutes at 37°C. Reactions were quenched with 1× SDS-PAGE loading buffer (4× stock), boiled for 5 minutes, and resolved by SDS-PAGE. Fluorescent signal was visualized in the rhodamine channel with a flatbed scanner.

### JCP174-BT competition labeling assay with recombinant enzyme

Recombinantly expressed HsAPT1, HsAPT2, and TgPPT1^WT^ or TgPPT1^S128A^ (100 ng) was resuspended in TBS buffer with 0.5% NP40 to a total volume of 19.8 μL. Indicated concentrations of inhibitor were added (100× stock in buffer), with the enzyme/inhibitor reaction incubated at 37°C for 60 minutes. After pre-incubation, JCP174-BT (100× stock in DMSO) was added to a final concentration of 1 μM. Samples were incubated at 37°C for 30 minutes. Reactions were quenched with 1× SDS-PAGE loading buffer (4× stock), boiled for 5 minutes, and resolved by SDS-PAGE. Fluorescent signal was visualized in the 4,4-difluoro-4-bora-3a,4a-diaza-*s*-indacene tetramethylrhodamine (BODIPY TMR) channel (ex: 543 nm, em: 569 nm) with a flatbed scanner. Equal protein loading was assessed via silver stain.

### Intact mammalian cell labeling with JCP174-BT

Cells were seeded at a density of 300,000 cells per well into a 6-well plate and allowed to adhere overnight. Intact adherent cells were pre-treated with different concentrations of JCP174 or palmostatin B for 1 hour at 37°C. Media was removed, cells were washed with PBS, and media containing 1 μM (unless otherwise indicated) of JCP174-BT was added to cells for an additional 1 hour at 37°C. Cells were washed with PBS and lysates were prepared by incubating pellets in TBS with 0.5% NP40 for 30 minutes on ice. Lysates were clarified via centrifugation at 13,000 rpm for 30 minutes, and total protein concentration was measured via BCA assay. Lysates were diluted to 20 μg total protein in a volume of 15 μL in TBS with 0.5% NP40, samples were prepared with 1× SDS-PAGE loading buffer (4× stock), boiled for 5 minutes, and resolved by SDS-PAGE. Fluorescent signal was visualized in the BODIPY TMR channel (ex: 543 nm, em: 569 nm) with a flatbed scanner. Where applicable, western blot analyses were performed with anti-HsAPT1 antibody (ProteinTech, rabbit polyclonal 16055-1-AP) (1:1000) and anti-GAPDH (Millipore, mouse monoclonal MAB374) (1:300).

### Intact parasite labeling with JCP174-BT

Intact extracellular *T*. *gondii* tachyzoites harvested by syringe lysis of heavily infected host-cells were pre-treated with different concentrations of JCP174 or palmostatin B for 15 minutes at 37°C. Parasites were washed and labeled with 1 μM (unless otherwise indicated) of JCP174-BT for 1 hour at 37°C. Parasites were washed with PBS and lysates were prepared by incubating pellets in Tris-buffered saline (TBS, 50 mM TRIS, 150 mM NaCl, pH 7.4) with 0.5% NP40 and 0.1% SDS for 30 minutes on ice. Lysates were clarified via centrifugation in a benchtop microcentrifuge at 13,000 rpm for 30 minutes, and total protein concentration was measured via BCA assay. Lysates were diluted to 20 μg total protein in a volume of 15 μL in TBS buffer with 0.5% NP40, samples were prepared with 1× SDS-PAGE loading buffer (4× stock), boiled for 5 minutes, and resolved by SDS-PAGE. Fluorescent signal was visualized in the BODIPY TMR channel (ex: 543 nm, em: 569 nm) with a flatbed Typhoon scanner.

### HsAPT1 IP

Intact mammalian U-2 OS cells were labeled with 1 μM JCP174-BT for 1 hour at 37°C. Cells were washed with PBS and lysates were prepared by incubating pellets in TBS buffer with 0.5% NP40 for 30 minutes on ice. Lysates were clarified via centrifugation at 13,000 rpm for 30 minutes, and total protein concentration was measured via BCA assay. To immunoprecipitate HsAPT1, 100 μg total protein was incubated with anti-HsAPT1 antibody (ProteinTech) in 250 μL of IP buffer (50 mM TRIS, 150 mM NaCl, pH 7.4, 0.5% NP40) for 15 minutes on ice, then immobilized on Protein G resin (40 μL of 50:50 slurry) with agitation overnight at 4°C. The reaction was washed 2 × 500 μL of IP buffer, 1 × 500 μL of IP buffer without NP40, aspirated dry and eluted by boiling with SDS-PAGE loading buffer. Input (10 μg), supernatant, and elution samples were resolved by SDS-PAGE. Fluorescent signal was visualized in the BODIPY TMR channel (ex: 543 nm, em: 569 nm) with a flatbed scanner before performing western blot analysis for HsAPT1. For western blot analysis, anti-HsAPT1 antibody (ProteinTech, rabbit polyclonal) (1:1000) primary and protein G HRP (Life technologies) (1:5000) secondary were used.

### General synthetic methods

All reagents were HPLC grade and used without further purification. The LC-MS data were acquired using an Agilent HPLC in tandem with an API 150 mass spectrometer (AppliedBiosystems/SCIEX) equipped with an electrospray interface. The general synthesis of the chloroisocoumarin scaffold has been described previously [12,29], and was followed with minor modifications.

### 2-(2-(but-3-yn-1-yloxy)-2-oxoethyl)-5-nitrobenzoic acid

A suspension of 4-nitrohomophthalic acid [30] (.53 g, 2.4 mmol) and 3-butyn-1-ol (5 equiv.) in toluene (2 mL/mmol) was heated to 70° C. A catalytic amount (~0.1 equiv.) of pTSOH was added and the reaction was stirred overnight until TLC indicated consumption of the starting material. The reaction was quenched with diluted sodium bicarbonate and extracted twice with ethyl acetate. The aqueous layer was acidified with 1M HCl and extracted twice with ethyl acetate. The combined organic layers were combined and dried with MgSO_4_ and concentrated *in vacuo* to afford the monoesterified product in 27% yield. ^1^H NMR (400 MHz, Chloroform-*d*) δ 8.98 (d, *J* = 2.5 Hz, 1H), 8.39 (dd, *J* = 8.4, 2.5 Hz, 1H), 7.52 (d, *J* = 8.4 Hz, 1H), 4.24 (t, *J* = 6.7 Hz, 2H), 4.21 (s, 2H), 2.54 (td, *J* = 6.7, 2.7 Hz, 2H), 2.01 (t, *J* = 2.7 Hz, 1H); ^13^C NMR (100 MHz, Chloroform-*d*) δ 170.1, 170.0, 147.4, 143.5, 133.9, 130.1, 127.7, 127.0, 79.9, 70.2, 63.1, 40.6, 19.0.

### 3-(3-Butynoxy)-4-chloro-7-nitro-isocoumarin

PCl_5_ (3 equiv.) was added to a solution of the monoesterified product (.15 g, .54 mmol) in toluene (7 mL) and solution was stirred overnight at 70° C. TLC (20% EtOAc/Hexanes) indicated complete consumption of the starting material. The reaction mixture was cooled and diluted with ethyl acetate and extracted with sodium bicarbonate. The aqueous layer was extracted 2× with ethyl acetate. The combined organic layers were washed with sodium bicarbonate and brine and subsequently dried with MgSO_4_ and concentrated *in vacuo*. The product was purified using silica column chromatography (5–20% EtOAc/Hexanes) to afford a yellow solid (30% yield). ^1^H NMR (400 MHz, Chloroform-*d*) δ 9.04 (d, *J* = 2.4 Hz, 1H), 8.53 (dd, *J* = 9.0, 2.3 Hz, 1H), 7.84 (d, *J* = 9.0 Hz, 1H), 4.56 (t, *J* = 6.8 Hz, 2H), 2.75 (td, *J* = 6.8, 2.7 Hz, 2H), 2.05 (t, *J* = 2.6 Hz, 1H).

### 3-(3-Butynoxy)-4-chloro-7-amino-isocoumarin (JCP174)

The nitro-isocoumarin product (.055 g, .19 mmol) was suspended in 4:1 EtOH:H_2_O (5 mL) and Fe (s) was added (10 equiv.) followed by 8 μL concentrated HCl. The mixture was refluxed at 80° C until disappearance of the starting material by TLC, approximately 1 hour. The mixture was cooled, diluted with EtOAc, and filtered through a pad of celite. The filtrate was washed with brine, dried with MgSO_4_ and concentrated *in vacuo*. The crude mixture was purified by reversed-phase HPLC (20% ACN/H_2_O +0.1% TFA to 43% ACN/H_2_O +0.1% TFA) to afford a yellow solid (54% yield after HPLC). ^1^H NMR (400 MHz, DMSO-*d*_6_) δ 7.44 (d, *J* = 8.6 Hz, 1H), 7.31 (d, *J* = 2.3 Hz, 1H), 7.19 (dd, *J* = 8.6, 2.4 Hz, 1H), 4.32 (t, *J* = 6.4 Hz, 2H), 2.90 (t, *J* = 2.6 Hz, 1H), 2.66 (td, *J* = 6.4, 2.6 Hz, 2H).

### JCP174-BODIPY TMR

JCP174-alkyne (9.0 μmol) was solubilized in 100 μL degassed MeOH. An equivalent volume of BODIPY-TMR-azide dissolved in dioxane was added, followed by a catalytic amount (~0.1 equiv) of TBTA and tetrakis(acetonitrile)copper(I) hexafluorophosphate. The mixture was stirred at room temperature for 3 h until complete consumption of the starting material was observed by TLC (5% MeOH/DCM, 3 drops of triethylamine). The reaction was diluted with DCM and MeOH and concentrated *in vacuo*. The compound was purified by silica column chromatography (1% MeOH/DCM) and further purified by reversed-phase HPLC to yield 0.6 mg JCP174-BODIPY TMR (9% yield). ^1^H NMR (600 MHz, Acetonitrile-*d*_3_) δ 7.82 (d, *J* = 8.9 Hz, 2H), 7.57 (s, 1H), 7.46 (d, *J* = 8.6 Hz, 1H), 7.33 (d, *J* = 2.0, 1H), 7.33 (s, 1H), 7.15 (dd, *J* = 8.6, 2.5 Hz, 1H), 7.03 (d, *J* = 4.0 Hz, 1H), 6.98 (d, *J* = 8.9 Hz, 2H), 6.58 (d, *J* = 3.8 Hz, 1H), 6.40 (t, *J* = 4.0 Hz 1H), 4.48 (t, *J* = 6.5 Hz, 2H), 4.15 (t, *J* = 7.1 Hz, 2H), 3.83 (s, 3H), 3.10–3.06 (m, 4H), 2.70 (t, *J* = 7.2 Hz, 2H), 2.48 (s, 3H), 2.27 (t, *J* = 7.3 Hz, 2H), 2.22 (s, 3H), 1.88 (m, 2H).

## Supporting information

S1 Fig Purification of HsAPT1 and HsAPT2. Overloaded Coomassie stained SDS-PAGE gel of purified recombinant HsAPT1 and HsAPT2 protein.
(DOCX)

S2 Fig Synthesis of JCP174-BT. Click reaction between JCP174-alk and BODIPY-TMR azide to generate the ABP JCP174-BT.
(DOCX)

S3 Fig 4-NPO activity assay with JCP174-BT. Dose-response curves for HsAPT1 and HsAPT2 recombinant enzyme with JCP174-BT in the 4-NPO esterase activity assay.
(DOCX)

S4 Fig FP-rho labeling of matched cancer cell lines. Mammalian cell lysates were labeled with FP-rho and resolved via SDS-PAGE. Pairs of oncogenic cell lines from derived from three tissue types (carcinomas of the breast, ovary and prostate) were chosen to contrast low metastatic/aggressive potential (MCF7, OVCAR-3, and LNCaP) versus high metastatic potential (MDA-MB-231, SKOV-3, and PC-3) [25]. For each oncogenic pair, wedges indicate low to high metastatic potential. FP-rho fluorescent signal was visualized with a flatbed scanner (top panel). Total HsAPT1 protein level was visualized by western blot (middle panel). GAPDH was used as a loading control (lower panel).
(DOCX)

S1 Table IC_50_ values of JCP174 and palmostatin B over time in the 4-NPO esterase assay. (DOCX)

S2 Table RMA-normalized mRNA expression data of HsAPT1 and HsAPT2 for cancer cell lines from the Cancer Cell Line Encyclopedia (CCLE)[26]. (DOCX)
