# Supplementary material for: Development of an activity-based probe for acyl-protein thioesterases
Source: PLoS One. 2018 Jan 24;13(1):e0190255. doi: 10.1371/journal.pone.0190255 (PMC5783350; doi:10.1371/journal.pone.0190255)
Supplement: S1 Fig — Overloaded Coomassie stained SDS-PAGE gel of purified recombinant HsAPT1 and HsAPT2 protein. (DOCX) [file pone.0190255.s001.docx]

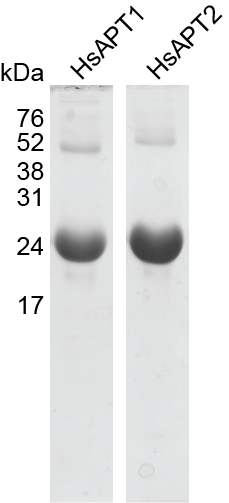


**S1 Fig. Purification of HsAPT1 and HsAPT2.** Overloaded Coomassie stained SDS-PAGE gel of purified recombinant HsAPT1 and HsAPT2 protein.
