# Supplementary material for: Development of an activity-based probe for acyl-protein thioesterases
Source: PLoS One. 2018 Jan 24;13(1):e0190255. doi: 10.1371/journal.pone.0190255 (PMC5783350; doi:10.1371/journal.pone.0190255)
Supplement: S2 Fig — Click reaction between JCP174-alk and BODIPY-TMR azide to generate the ABP JCP174-BT. (DOCX) [file pone.0190255.s002.docx]

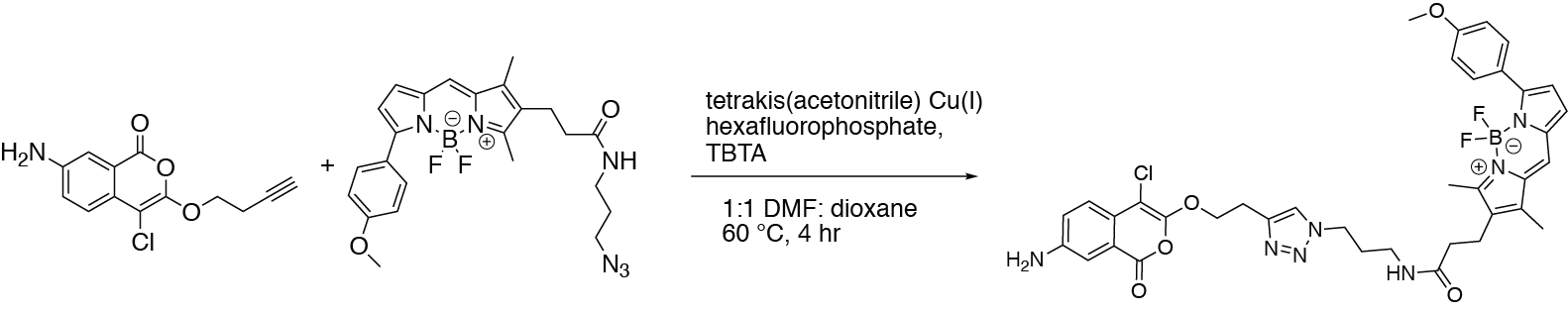


**S2 Fig. Synthesis of JCP174-BT.** Click reaction between JCP174-alk and BODIPY-TMR azide to generate the ABP JCP174-BT.
