# Supplementary material for: Development of an activity-based probe for acyl-protein thioesterases
Source: PLoS One. 2018 Jan 24;13(1):e0190255. doi: 10.1371/journal.pone.0190255 (PMC5783350; doi:10.1371/journal.pone.0190255)
Supplement: S3 Fig — Dose-response curves for HsAPT1 and HsAPT2 recombinant enzyme with JCP174-BT in the 4-NPO esterase activity assay. (DOCX) [file pone.0190255.s003.docx]

**S3 Fig. 4-NPO activity assay with** **JCP174-BT.** Dose-response curves for HsAPT1 and HsAPT2 recombinant enzyme with JCP174-BT in the 4-NPO esterase activity assay.
