# Supplementary material for: Development of an activity-based probe for acyl-protein thioesterases
Source: PLoS One. 2018 Jan 24;13(1):e0190255. doi: 10.1371/journal.pone.0190255 (PMC5783350; doi:10.1371/journal.pone.0190255)
Supplement: S4 Fig — Mammalian cell lysates were labeled with FP-rho and resolved via SDS-PAGE. Pairs of oncogenic cell lines from derived from three tissue types (carcinomas of the breast, ovary and prostate) were chosen to contrast low metastatic/aggressive potential (MCF7, OVCAR-3, and LNCaP) versus high metastatic potential (MDA-MB-231, SKOV-3, and PC-3) [25]. For each oncogenic pair, wedges indicate low to high metastatic potential. FP-rho fluorescent signal was visualized with a flatbed scanner (top panel). Total HsAPT1 protein level was visualized by western blot (middle panel). GAPDH was used as a loading control (lower panel). (DOCX) [file pone.0190255.s004.docx]

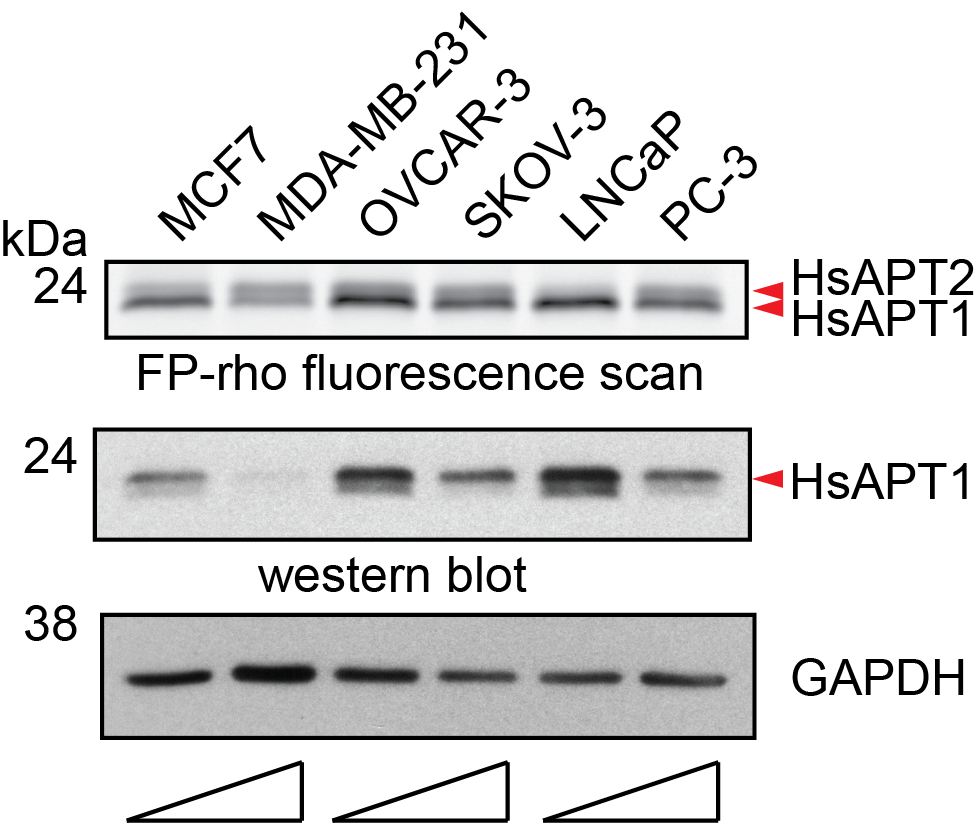


**S4 Fig. FP-rho labeling of matched cancer cell lines.** Mammalian cell lysates were labeled with FP-rho and resolved via SDS-PAGE. Pairs of oncogenic cell lines from derived from three tissue types (carcinomas of the breast, ovary and prostate) were chosen to contrast low metastatic/aggressive potential (MCF7, OVCAR-3, and LNCaP) versus high metastatic potential (MDA-MB-231, SKOV-3, and PC-3) [25]. For each oncogenic pair, wedges indicate low to high metastatic potential. FP-rho fluorescent signal was visualized with a flatbed scanner (top panel). Total HsAPT1 protein level was visualized by western blot (middle panel). GAPDH was used as a loading control (lower panel).
