# Supplementary material for: Development of an activity-based probe for acyl-protein thioesterases
Source: PLoS One. 2018 Jan 24;13(1):e0190255. doi: 10.1371/journal.pone.0190255 (PMC5783350; doi:10.1371/journal.pone.0190255)
Supplement: S1 Table — (DOCX) [file pone.0190255.s005.docx]

**S1 Table. IC_50_ values of JCP174 and palmostatin B over time in the 4-NPO esterase assay**

| **Compound** | **Preincubation time (hr)** | **HsAPT1 IC_50_ (μM)** | **HsAPT2 IC_50_ (μM)** |
| --- | --- | --- | --- |
| JCP174 | 0.5 | 1.7 | 0.75 |
|  | 2 | 0.27 | 0.34 |
|  | 6 | 0.16 | 0.23 |
| Palmo B | 0.5 | 0.31 | 0.32 |
|  | 2 | 2.5 | 0.70 |
|  | 6 | 6.8 | 1.6 |
