# Supplementary material for: Development of an activity-based probe for acyl-protein thioesterases
Source: PLoS One. 2018 Jan 24;13(1):e0190255. doi: 10.1371/journal.pone.0190255 (PMC5783350; doi:10.1371/journal.pone.0190255)
Supplement: S2 Table — (DOCX) [file pone.0190255.s006.docx]

**S2 Table. RMA-normalized mRNA expression data of HsAPT1 and HsAPT2 for cancer cell lines from the Cancer Cell Line Encyclopedia (CCLE)[26]**

| **Cell line** | **HsAPT1 normalized expression** | **HsAPT2 normalized expression** |
| --- | --- | --- |
| **MCF7** | 11.7 | 8.1 |
| **MDA-MB-231** | 10.4 | 8.5 |
| **OVCAR-3** | 11.3 | 8.4 |
| **SKOV-3** | 11.4 | 8.3 |
| **LNCaP** | 11.5 | 7.3 |
| **PC-3** | 9.7 | 8.7 |
